# Transcriptomic analysis of gonadal development in parasitic and non-parasitic lampreys (*Ichthyomyzon* spp.), with a comparison of genomic resources in these non-model species

**DOI:** 10.1093/g3journal/jkab030

**Published:** 2021-02-12

**Authors:** Nisha Ajmani, Tamanna Yasmin, Margaret F Docker, Sara V Good

**Affiliations:** 1 Department of Biological Sciences, University of Manitoba, Winnipeg, Canada; 2 Department of Biology, University of Winnipeg, Winnipeg, Canada

**Keywords:** lamprey, sex differentiation, non-model organism, RNA sequencing, transcriptome, gene ontology enrichment, reference-based approach

## Abstract

Lampreys are jawless fishes that diverged ∼550 million years ago from other vertebrates. Sequencing of the somatic and the germline genomes of the sea lamprey (*Petromyzon marinus*) in 2013 and 2018, respectively, has helped to improve our understanding of the genes and gene networks that control many aspects of lamprey development. However, little is known about the genetic basis of gonadal differentiation in lampreys, partly due to the prolonged period during which their gonads remain sexually indeterminate. We performed RNA-sequencing on gonadal samples from four chestnut lamprey (*Ichthyomyzon castaneus*) and six northern brook lamprey (*I. fossor*) to identify differentially expressed genes (DEG’s) and pathways associated with transcriptomic differences in: (1) larvae during early gonadal differentiation *versus* definitive females (*i.e.*, with oocytes in the slow cytoplasmic growth phase); and (2) females *versus* definitive males undergoing spermatogonial proliferation. We compared the mapping percentages of these transcriptomes to the two available sea lamprey reference genomes and three annotation files (Ensembl and UCSC for the somatic genome and SIMRbase for the germline genome). We found that mapping the RNA-seq reads to the germline genome gave superior results and, using Trinotate, we provided new putative annotations for 8161 genes in the somatic assembly and 880 genes for the germline assembly. We identified >2000 DEG’s between stages and sexes, as well as biological pathways associated with each. Interestingly, some of the upregulated genes (*e.g.*, DEG’s associated with spermiation) suggest that changes in gene expression can precede morphological changes by several months. In contrast, only 81 DEG’s were evident between the chestnut lamprey (that remains sexually immature during an extended post-metamorphic parasitic feeding phase) and the nonparasitic northern brook lamprey (that undergoes sexual maturation near the end of metamorphosis), but few replicates were available for comparable stages and sexes. This work lays the foundation for identifying and confirming the orthology and the function of genes involved in gonadal development in these and other lamprey species across more developmental stages.

## Introduction

Lampreys, one of the two extant lineages of jawless vertebrates, are important model organisms for the study of early vertebrate evolution (Shimeld and Donoghue 2012; Docker *et al.* 2015; McCauley *et al.* 2015). They diverged from the rest of the vertebrate lineage between 500 and 600 million years ago (mya)(Dos Reis *et al.* 2015; Kumar *et al.* 2017), either after the two rounds (2R) of whole genome duplication (WGD) that occurred in early vertebrate evolution (Kuraku 2008; Sacerdot *et al.* 2018), or more likely after 1 R (Smith and Keinath 2015; Smith *et al.* 2018; Simakov *et al.* 2020). Sequencing of the somatic and the germline genomes of the sea lamprey (*Petromyzon marinus*) in 2013 and 2018, respectively, were important milestones for comparative vertebrate genomics (Smith *et al.* 2013; Smith *et al.* 2018). Nevertheless, the sea lamprey genome exhibits several features that complicate genomic studies. In addition to containing high levels of repetitive sequence (60% high-identity repeats) and being GC rich (Smith *et al.* 2018), lampreys undergo a programmed genome rearrangement, during which ∼20% of the germline genome is jettisoned from somatic cell lineages after ∼3 days post-fertilization (Bryant *et al.* 2016; Timoshevskiy *et al.* 2017; Smith *et al.* 2018). Moreover, lampreys exhibit many lineage-specific changes (Smith *et al.* 2018), owing to them having diverged from other vertebrates during a period of extensive paralog gene loss (following-1R) as well as during the expansion of vertebrate signalling networks involved in nervous, immune, and endocrine functions (Hufton *et al.* 2008; Kassahn *et al.* 2009; Canestro *et al.* 2013).

These genomic resources have been of great use in helping to improve our understanding of genes and gene networks that control many aspects of sea lamprey biology (Hockman *et al.* 2019; Jones *et al.* 2019; Parker *et al.* 2019; see review by York *et al.* 2019). However, relatively little is known about the genes involved in lamprey reproduction. In addition to the idiosyncrasies of their genome, understanding the genetic basis of lamprey sex determination (*i.e.*, the “master switch” that determines the ultimate fate of the gonad) and sex differentiation (the process by which the undifferentiated gonad develops into a recognizable ovary or testis) is complicated by their long and complex life cycle, the prolonged period during which their gonads remain histologically undifferentiated, and uncertainties regarding which sex steroids are physiologically relevant (see review by Docker *et al.* 2019). Environmental sex determination (ESD) has been proposed in lampreys, but evidence for ESD is equivocal; genes involved in sex determination in the “lower vertebrates” (*i.e.*, reptiles and non-amniotes) are far less conserved than they are in birds and mammals, and whether there is a genetic component to sex determination is lampreys is unknown (Docker *et al.* 2019). In contrast, many of the genes involved in the sex differentiation process tend to be conserved among vertebrates (Siegfried 2010), and initial studies suggest that at least some of the same genes are involved in gonadal development in lampreys (Spice *et al.* 2014; Khan 2017; see below).

There are at least 41 extant species of lampreys, all with a long larval stage spent burrowed in river sediments, where they grow slowly filter-feeding on detritus and algae (Potter *et al.* 2015). After ∼4–7 years as larvae, they undergo a pronounced metamorphosis during which the eye and the characteristic lamprey oral disc appear. In 18 species (including the sea lamprey), metamorphosis is followed by a sexually immature juvenile stage, which feeds parasitically on ray-finned fishes or other aquatic vertebrates for few months to a few years. Sexual maturation is initiated at the end of the parasitic feeding stage, and the lampreys embark on a non-trophic upstream migration to the spawning grounds, and die after a single spawning season (Potter *et al.* 2015). The remaining species are nonparasitic (*i.e.*, they do not feed at all following metamorphosis), and they show an accelerated sexual maturation, and spawn and die in their natal stream within 6–10 months of metamorphosis (Docker 2009).

**Figure 1:**
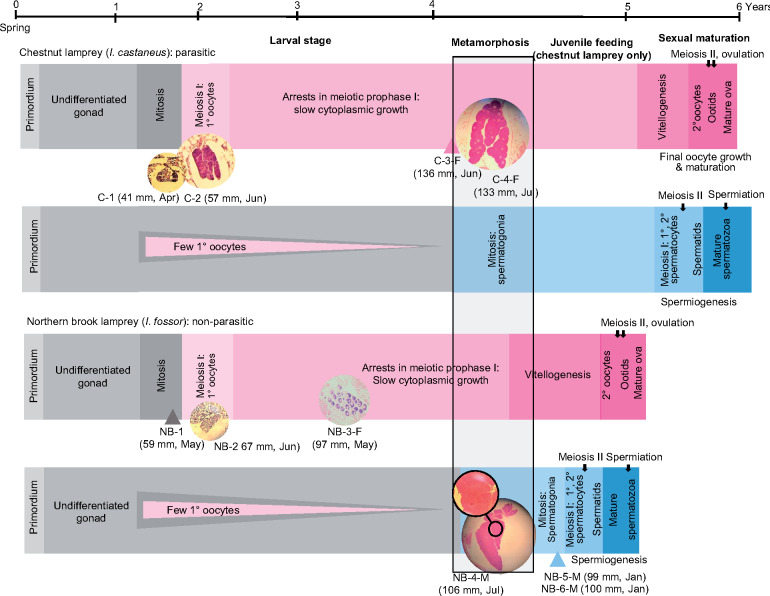
Schematic representation of lamprey gonadal development in chestnut and northern brook lampreys; the chestnut lamprey delays sexual maturation while feeding parasitically after metamorphosis, while the northern brook lamprey omits the post-metamorphic feeding stage and initiates sexual maturation during the later stages of metamorphosis. Pink and blue represent ovarian and testicular developmental stages, respectively; gray represents the period of sexual indeterminacy, which is particularly prolonged in future males. Histological images on the timeline illustrate where each of the 10 samples used in this study fall in the developmental process. Adapted from Docker *et al.* (2019).

In all species, there is a prolonged period during which the single elongated gonad remains histologically undifferentiated, and lampreys are sometimes said to pass through an initial female or intersexual stage, because at least a few small oocytes appear in most larvae regardless of future sex (Hardisty 1965a,b; Docker *et al.* 2019; Figure 1). Meiosis and oocyte growth are more synchronized and extensive in females, and ovarian differentiation is completed during the larval stage (at ∼1–3 years of age). Ovarian differentiation generally occurs earlier in nonparasitic species, and this usually results in the elaboration of fewer oocytes that presages the lower number of eggs evident at maturity in nonparasitic lampreys compared to the large-bodied parasitic species (Hardisty 1970; Docker 2009; but see Spice and Docker 2014). In all lampreys, testicular differentiation does not occur until the onset of metamorphosis.

A candidate gene approach has been used to test whether some of the genes implicated in gonadal differentiation in other vertebrates show sex- or stage-specific patterns of expression in lampreys. Spice *et al.* (2014) examined expression of eight candidate genes during ovarian differentiation in parasitic chestnut and nonparasitic northern brook lampreys (*Ichthyomyzon castaneus* and *I. fossor*, respectively), and Mawaribuchi *et al.* (2017) tested for up-regulation of *DMRT1* (which has been linked to testicular development in other vertebrates) in male *versus* female Far Eastern brook lamprey (*Lethenteron reissneri*), another nonparasitic species. Khan (2017) compared expression of seven candidate genes in male *versus* female sea lamprey (for which a nonparasitic counterpart does not exist) and among stages of testicular differentiation. However, a candidate gene approach limits us to a relatively small number of genes that have been associated with sex differentiation in other vertebrates.

A transcriptomic approach would be valuable to better identify the range of genes up- and down-regulated during gonadal development, but it is not clear to what extent sea lamprey genomic resources can be used for work on other lamprey species. Here, we performed transcriptomic sequencing on gonadal samples of the chestnut lamprey and the northern brook lamprey, during the early stages of sex differentiation and in differentiated females and males. The *Ichthyomyzon* and *Petromyzon* genera belong to the same family and subfamily (Potter *et al.* 2015), and molecular clock estimates suggest that they diverged ∼7–8 mya (Bartels *et al.* 2012). *Ichthyomyzon* species are more closely related to the sea lamprey than other lamprey taxa (*i.e., Lampetra*, *Entosphenus*; Kumar *et al.* 2017) for which the sea lamprey reference genome has been used to identify single nucleotide polymorphisms (SNPs) in regions of interest (*e.g.*, Hess *et al.* 2013; Mateus *et al.* 2013; Rougemont *et al.* 2017; Hess *et al.*, 2020).

The current study had the following three objectives: (1) compare the mapping statistics of the RNA-seq reads to both the somatic and the germline reference genomes and integrate new functional annotations of genes derived from implementation of the Trinotate pipeline to existing annotations at Ensembl and UCSC (for the somatic genome) and SIMRbase (for the germline genome); (2) using only the superior germline reference genome and annotation, identify if there are global transcriptomic changes across gonadal stage, sex, and species (*i.e.*, parasitic *versus* nonparasitic lampreys); and (3) identify the top pathways and genes associated with each gonadal stage.

## Materials and methods

### Sample collection

Chestnut and northern brook lampreys were collected in 2011 and 2012 (Spice 2013; Spice *et al.* 2014), using a backpack electrofisher (Smith-Root LR-24) in accordance with provincial and federal collection permits (SCP 05-11/15-12 and SECT 73 SARA C&A 11-012/12-009, respectively). The chestnut lamprey were collected from the Rat River in St. Malo, Manitoba, while the northern brook lamprey were collected from the Birch River near Prawda, Manitoba, and McKinnon Creek near Sault Ste. Marie, Ontario. Lampreys were sacrificed according to the University of Manitoba Animal Use Protocol F11-019, and gonads were removed after ensuring the death of the individuals. Most of the lampreys were killed within several hours of collection, but two individuals (NB-5-M and NB-6-M) were held in aquaria in the Department of Biological Sciences Animal Holding Facility until they completed metamorphosis (Table 1). Metamorphosis, which occurs during late summer and early fall, is divided into seven stages based on changes in several key external morphological features (*e.g.*, appearance of the eye, and remodeling of the mouth (Manzon *et al.* 2015)).

**Table 1:** Details on individuals and their gonadal stages used in this study. Samples are labelled as “C” and “NB” for chestnut (*Ichthyomyzon castaneus*) and northern brook lamprey (*I. fossor*), respectively, and in sequence according to the degree of gonadal development. The “F” and “M” suffixes are for definitive females and males, and no suffix indicates undifferentiated individuals or those in the early stages of differentiation. Life stages are labelled as “L” for larvae and “MET” for metamorphosing (stages 1–7) or metamorphosed (C) individuals (see text). Gonadal characteristics are illustrated in Figure 1 and described in detail in Docker *et al.* (2019).

Species	Sample ID	Date of sacrifice	Length (mm)	Life stage	Sex	Gonadal characteristics	
Chestnut lamprey	C-1	Apr 30, 2012	41	L	Early	Cysts of undifferentiated germ cells following onset of mitosis		
	C-2	Jun 7, 2012	57	L	Early	Onset of meiosis I, showing early stages of oocyte differentiation (*i.e.*, prior to rapid phase of cytoplasmic growth); likely a future female, but a small number of primary oocytes also appear in a large proportion of the presumptive male lamprey larvae		
	C-3-F	Jun 7, 12	136	MET (2/3)	F	Differentiated ovary in cytoplasmic growth stage with non-vitellogenic oocytes		
	C-4-F	Jul 27, 2011	133	MET (2/3)	F	Differentiated ovary in cytoplasmic growth stage with non-vitellogenic oocytes		
Northern brook lamprey	NB-1	May 25, 2011	59	L	Early	Cysts of undifferentiated germ cells following onset of mitosis		
	NB-2	Jun 13, 2012	67	L	Early	Early stage of meiosis I, with oocytes in primary growth stage; presumptive but not definitive female (see C-2)		
	NB-3-F	May 25, 2011	97	L	F	Differentiated ovary in cytoplasmic growth stage with non-vitellogenic oocytes		
	NB-4-M	Jul 27, 2011	106	MET (4/5)	M	Differentiated testis showing spermatogonial proliferation		
	NB-5-M	Jan 21, 2012	99	MET (C)	M	Differentiated testis showing spermatogonial proliferation		
	NB-6-M	Jan 21, 2012	100	MET (C)	M	Differentiated testis showing spermatogonial proliferation		

A section of the gonad was fixed for histological examination, and the remaining gonadal tissue was placed in liquid nitrogen and stored at –80°C. Total RNA was subsequently extracted using the Qiagen RNeasy Mini Kit, following the manufacturer’s instructions, and genomic DNA was removed using the RNase-free DNase Set (Qiagen). Total concentration of RNA was measured, and RNA was kept at –80°C until use (Spice *et al.* 2014).

### Gonadal developmental stages

Ten individuals representing three key stages in lamprey gonadal differentiation were selected for RNA sequencing: (1) “early differentiation” in which larvae were either undergoing mitotic proliferation of undifferentiated germ cells (C-1, NB-1) or the early stages of meiosis I (C-2 and NB-2); (2) differentiated females (C3-F, C4-F, NB-3-F) showing oocytes in the slow cytoplasmic (pre-vitellogenic) growth phase; and (3) males with differentiated testes undergoing spermatogonial proliferation (NB-4-M, NB-5-M, NB-6-M) (see Table 1, Figure 1). C-2 and NB-2 are likely presumptive females, but, because meiosis also occurs during the larval stage in at least some future male lampreys, gonads with a small number of primary oocytes are generally not characterized as ovaries until the oocytes progress to the cytoplasmic growth phase (Docker *et al.* 2019). Note that testicular differentiation is initiated in the early stages of metamorphosis, while the ovary does not undergo dramatic changes at the onset of metamorphosis (Figure 1).

### Library preparation and RNA sequencing

Total RNA from three samples (C-1, C-2, and NB-2) was sent to the Hussman Institute for Human Genomics (Miami, Florida); the other samples were sent to the Oklahoma Medical Research Foundation (Oklahoma City, Oklahoma) (Spice 2013). Messenger RNA (mRNA) was isolated using a poly-A or poly-T step, respectively, and non-normalized libraries were prepared using the Illumina TruSeq DNA Kit and EpicentreScriptSeq Kit (San Diego, California). Sequencing was performed using 100 base pair (Hussman) and 75 base pair (Oklahoma) paired-end sequencing on an Illumina Hi-Seq 2000.

### Quality control and pre-processing of reads

The resulting RNA-Seq paired-end (PE) reads were checked for quality using FASTQC (Andrews 2010) and adapter sequences trimmed with Trimmomatic (v 0.32) (Bolger *et al.* 2014) (parameters: ILLUMINACLIP: TruSeq3-PE-2.fa : 2:15:10 LEADING : 5 TRAILING : 5 SLIDINGWINDOW : 4:5 MINLEN : 50); reads shorter than 50 bp were removed and the remaining reads were “groomed” using FASTQ Groomer (Blankenberg 2010).

### Mapping and assembling of RNA seq reads

*Somatic:* The current somatic genome for sea lamprey is Pmarinus_7.0, (Jan, 2011) which spans 647.38 Mbp as described in Smith *et al.* (2013), and is available at NCBI (https://www.ncbi.nlm.nih.gov/assembly/GCA_000148955.1/), Ensembl (https://uswest.ensembl.org/Petromyzon_marinus/Info/Index), and UCSC (http://genome.ucsc.edu/). PE groomed reads were aligned to the somatic genome of sea lamprey available at Ensembl (v 87.7) using TopHat (Trapnell *et al.* 2012), with a mean inner distance between mate pairs of 220 bp (estimated from the data) and a final alignment mismatch score of 4, to account for divergence between *Ichthyomyzon* and *Petromyzon* genomes. From the TopHat output, only the accepted hits file (in BAM format) was carried forward to the next step. Next, the *htseq-count union* script (Anders *et al.* 2015) was employed to calculate the mapping statistics based on the somatic reference genome (as above) with annotation files available from both Ensembl and UCSC (based on Smith *et al.* 2013). Briefly, the Ensembl annotation includes annotations for 13,114 genes of which 10,415 are protein coding genes (Ensembl database 99.7), while that at UCSC includes 26,046 protein coding variants of 22,297 genes. Reads mapping to a singular genomic region and gene as defined by the annotation file are counted as reads to “features,” those mapping to both genic and nongenic regions are called “ambiguous,” those mapping to multiple different genes (*e.g.*, repetitive regions) are classified as “alignment not unique,” while those mapping to regions of the genome with no annotation are grouped as “no feature.” It is worth noting that the sum of all counts will not be equal to the number of reads, because multiple or overlapping alignments are scored multiple times. Using reference based transcriptomic analyses is limited by the fact that reads mapping to “no feature” have no associated ID, even though they are likely to contain novel genes, and are not analysed further. To allow comparison of the mapping statistics of reads to the somatic *vs* the germline assemblies (see below), the number of reads mapping to the somatic genome using the UCSC annotation file were also quantified using RseQC (v. 3.0.1) (Wang *et al.* 2012).

**Figure 2:**
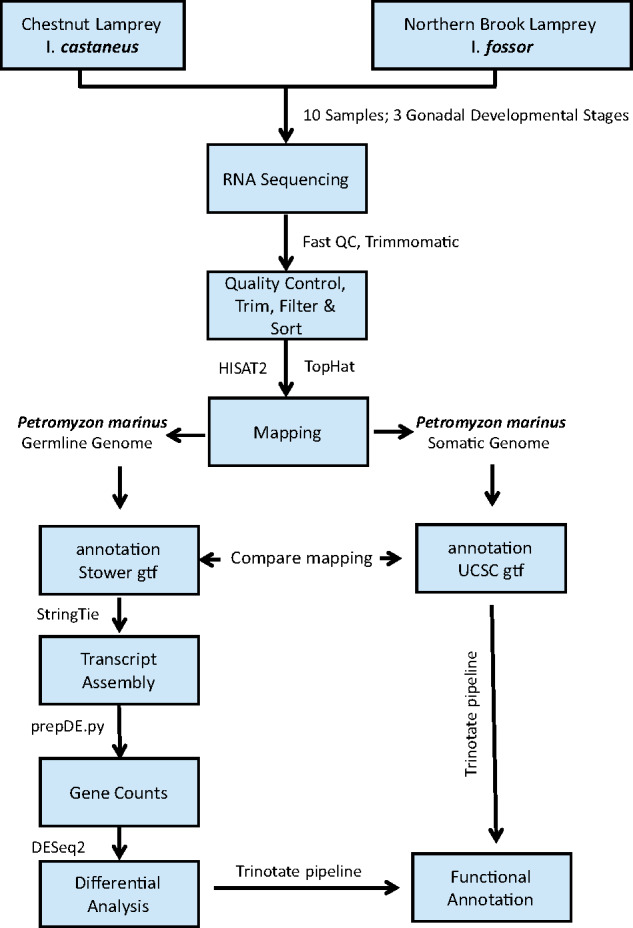
Transcriptomics data analysis pipeline employed in this study for the reference-based transcriptome assembly and annotation using both the somatic and the germline reference genomes available at UCSC, Ensembl, and SIMRbase (see text).

*Germline:* An assembly of the sea lamprey germline genome (based on testes of spawning adults), which harbours ∼20% more DNA than somatic cells, was released in 2018 (Smith *et al.* 2018). We used the fastA sequence from this assembly (Pmar_germline 1.0) and associated gene annotation file available from SIMRbase (downloaded from https://genomes.stowers.org/organism/Petromyzon/marinus) to map the gonadal transcripts. The germline genome assembly spans 1007.68 Mbp and the accompanying SIMRbase gene annotation file contains 20,950 protein coding genes. Trimmed PE fastq files were aligned to the germline genome using HISAT2 (v 2.1.0) (hisat2 –p8 –dta –x index_file-1 read_pair1.fastq -2 read_pair2.fastq –S output.sam) (Pertea *et al.* 2016); the resulting SAM files were indexed, sorted, and converted to BAM format using Samtools (v 1.9, http://www.htslib.org/). Mapped reads were assembled into transcripts using the germline annotation file available at SIMRbase using the StringTie (v 2.0) algorithm and the -e parameter (Pertea *et al.* 2016). Finally, mapping statistics of each sample were generated with RSeQC (v 3.0.1) (Wang *et al.* 2012) (Figure 2).

### Functional annotation of genes, and generation of merged annotation tables linking Ensembl and UCSC gene names for the somatic and the germline genes

*Somatic:* Of the 10,415 protein coding genes annotated at Ensembl and the 22,297 unique protein coding genes we identified at UCSC (see Results), only 3414 and 3617 had gene names associated with them. However, the two databases use different gene identifiers, and most of the genes (on both databases) are not associated with the candidate gene names. Therefore, to assign putative gene names and gene ontologies for more genes, we employed Trinotate (v 3.0) (http://trinotate.github.io) (Bryant *et al.* 2017) to generate an automated functional annotation of all of the transcripts available at UCSC. The Trinotate pipeline uses a number of well-referenced methods to assign functional annotations, including homology searches to both protein and nucleotide databases (BLAST+/SwissProt) and protein domain identification (HMMER/PFAM), and it leverages annotation databases (GO). The individual transcript reports were loaded into the SQLite database to generate an annotation report for the whole transcriptome. To link our three sources of information (Ensembl, UCSC, and Trinotate), we extracted the Ensembl gene IDs from the transcript fileavailable at UCSC and then merged the three databases with the gene count data for analyses (Figure 2).

*Germline:* For the germline annotation at SIMRbase, there are putative gene names available for 17,883 of the 20,950 genes (assigned using MAKER, as per Smith *et al.* 2018). In addition, we submitted all protein coding transcripts identified in the germline genome (available at SIMRbase) to the Trinotate pipeline for functional annotation and assignment of GO terms, and then merged the Trinotate gene names with those assigned by Smith *et al.* (2018). To further link transcript ID’s from the somatic and germline assemblies, we performed a blast-all against all on the coding sequence transcript file for the somatic and germline files, and included all somatic-gonad transcript alignments with a Bit Score >50.

### Differential gene expression analyses

Although the chestnut and northern brook lampreys differ in their life history following completion of metamorphosis, preliminary analyses of global transcriptomic differences for our samples showed strong similarities within each gonadal stage and sex regardless of post-metamorphic feeding type (see Results). Thus, to provide biological replicates for the purposes of differentially expressed genes (DEG) analyses, samples were pooled into the three groups identified above, regardless of species: (1) early differentiation (C-1, C-2, NB-1, and NB-2); (2) differentiated females (C3-F, C4-F, and NB-3-F); and (3) differentiated males (NB-4-M, NB-5-M, and NB-6-M) (see Table 1, Figure 1). Pairwise comparisons were made between the “early” larvae (which included two presumptive females) and differentiated females (1 *vs* 2) and between differentiated females and males (2 *vs* 3).

*Germline:* Genes for which there were >10 counts across all samples were used as input to test for evidence of DEGs using the algorithm in DESeq2 (design = ∼stage) followed by pairwise contrasts (early *vs* F and F *vs* M) (Anders *et al.* 2015). The gene/transcript counts were obtained from the stringtie output using a python script (https://github.com/gpertea/stringtie/blob/master/prepDE.py). DEG’s at an FDR < 0.01 were tabulated and used for the GO enrichment analyses. Additionally, an unsupervised heat map and Principal Component Analyses (PCA) plot of the relationship of global gene expression among samples was generated, and DEG’s were visualized with volcano plots, highlighting those genes having a log2fold change > 4 and a *P* < 10^−14^. Differential expression analysis was conducted in R platform v 4.0.2 using the packages DESeq2 (Love *et al.* 2014) and Enhanced Volcano (Blighe *et al.* 2018).

*Gene ontology enrichment:* Gene ontology enrichment was performed to identify pathways associated with early larvae, differentiated females, and differentiated males by taking the list of: (1) DEG’s that were downregulated (*i.e.*, associated with early differentiation) or (2) upregulated (associated with differentiated females) in the comparison of females to early larval samples, and those that were (3) down-regulated (associated with differentiated females) or (4) upregulated (associated with differentiated males) in the comparison of males to females. In this way, we aimed to identify genes and pathways associated with (a) mitosis/early meiosis in early differentiating gonads (see Figure 1), (b) ovarian development, and (c) testicular differentiation. These four lists of genes were submitted to Gorilla (Eden *et al.* 2009), to identify global pathways and associated GO terms using *Homo sapiens* as a model. Enrichment values were obtained for three GO domains: Biological Processes, Functions, and Component. To compare expression of specific genes associated with stage-specific processes, the normalized counts of genes showing strong stage-specific expression and associated with specific GO terms were extracted from the DESeq2 output and plotted in a heatmap using the on-line tool Morpheus (https://software.broadinstitute.org/morpheus/).

## Results and discussion

### Mapping and gene count comparison between somatic and germline genomes

#### Comparison of mapping statistics and gene counts for the somatic and germline reference genomes

*Somatic genome—comparison of UCSC and Ensembl annotation files:* On average, ∼1.6 million more reads mapped to features (genes) using the UCSC compared to the Ensembl annotation for the somatic genome; additionally, 8646 more genes were identified and 6594 more genes (16,168 *vs* 9574) had counts >10 (Supplementary Table S1). For the Ensembl annotation file, the HTseq output included counts for 13,114 genes, of which 10,593 were protein coding genes, and 6903 had an associated gene name (Supplementary Table S2a). For the UCSC annotation file, there were 22,297 protein coding genes (with ids beginning with PMZ_#) for which 19,191 had nonzero counts (Supplementary Table S2b). Collectively, this suggests that the somatic annotation file at UCSC is substantially larger and more detailed than that at Ensembl.

*Germline* vs *somatic reference genomes:* We then compared the mapping percentages of the 10 chestnut and northern brook lamprey gonad transcriptomes to either the somatic or the germline reference genomes using RSEQC (Table 2). Except for two samples (C-1 and NB-3-F), the number of reads mapping to the germline reference genome was at least two times greater than the number mapping to the somatic genome: ∼10–71 Mbp *vs* ∼6–23, and was greater than 30 million reads for all but these two samples (Table 2). The germline reference genome contains ∼300 Mbp more sequence than the somatic genome (10,007 Mbp *versus* 686 Mbp), and it is a substantially improved assembly with 12,061 scaffolds and 24,392 contigs (Contig N50 = 170,712 and Contig L50 = 1431) compared to the 25,005 scaffolds and 73,813 contigs (Contig N50 = 13,108 and Contig L50 = 14,492) present in the somatic genome.

**Table 2:** Mapping statistics and gene counts for the 10 *Ichthyomyzon* gonadal transcriptomes mapped to the somatic (SOMA) or germline (GERM) sea lamprey (*Petromyzon marinus*) reference genomes; the reference annotation file for the somatic genome was that available at UCSC (see text), and for the germline genome was that available at SIMRbase (https://genomes.stowers.org/). RNA-Seq was performed on four undifferentiated, three female (F), and three male (M) chestnut lamprey (*I. castaneus*, C) or northern brook lamprey (*I. fossor*, NB). The total number of reads, number of mapped reads, and percent of mapped reads were obtained from the bam.py script in RSeQC. The number of genes with nonzero and >10 counts per sample were generated from HTseq for the somatic assembly and from Stringtie/PredPE scripts for the germline assembly. Mapped reads are those that align to an annotated feature (*e.g.*, exon, gene) in the reference genome, while those that are unmapped contain reads that either align to an unannotated section of the reference genome or do not align to any portion of the genome. For the gene count data, the average number of reads per sample (average) as well as the number of genes identified across all samples (all samples) are also given.

Sample	Total reads	No. mapped reads		% mapped reads		Genes >0 counts		Genes >10 counts		% >0 counts	
		GERM	SOMA	GERM	SOMA	GERM	SOMA	GERM	SOMA	GERM	SOMA
***C-1***	34,201,424	9,807,291	9,656,978	29	28	18,166	15,094	12,929	8,174	87	68
***C-2***	114,063,922	32,800,544	12,808,172	29	11	19,425	14,480	15,906	9,098	93	65
***C-3-F***	147,855,768	71,066,894	10,644,862	48	7	19,205	15,017	15,950	11,126	92	67
***C-4-F***	139,081,313	61,174,674	17,261,170	44	12	19,243	15,765	15,826	11,490	92	71
***NB-1***	135,950,011	46,777,479	6,214,354	34	5	19,562	15,295	16,497	10,791	93	69
***NB-2***	109,857,031	30,417,303	5,747,982	28	5	18,297	14,573	13,778	9,163	87	65
***NB-3-F***	26,156,078	10,301,130	8,945,379	39	34	19,093	15,253	15,111	11,139	91	68
***NB-4-M***	152,882,912	65,073,704	22,674,294	43	15	19,125	14,726	15,528	11,180	91	66
***NB-5-M***	117,599,911	36,123,269	19,359,244	31	16	19,156	14,560	14,685	10,518	91	65
***NB-6-M***	121,627,377	48,590,166	9,599,236	40	8	19,188	15,184	16,065	10,824	92	68
**Average**	**109,927,575**	**41,213,245**	**12,291,167**	**36**	**14**	**19,046**	**14,995**	**15,228**	**10,350**	**91**	**67**
**All samples**						**20,521**	**19,191**	**19,695**	**16,168**	**98**	**86**

*Most of the genes present in the germline annotation file were expressed in our samples:* 18,166–19,562 of the 20,950 lamprey genes (87%–93%) had nonzero counts in each individual, and over all samples, 98% (20,521) of genes had nonzero counts and 94% (19,695) had more than 10 gene counts (Table 2, Supplementary Table S3). On the other hand, only 65%–71% of the 22,297 protein coding genes in the somatic genome had nonzero counts in an individuals and, overall, 86% had nonzero counts and 73% had more than 10 gene counts. This shows that even though the proportion of total reads that mapped to the reference germline assembly was < 50%, the sea lamprey genome can be effectively used to map reads from *Ichthyomyzon*. Cursory examination of the unmapped reads indicated that many of them were likely mitochondrial transcripts, unassembled repeats, or non-lamprey transcripts, but a full analysis of the percentage of unmapped reads falling into each category was not performed. The superior performance of read-mapping to the germline genome is likely also due to the implementation of the HiSat2 algorithm, compared to TopHat; HiSat2 is known to be robust to single base pair differences between samples and reference genome, and may have further improved cross-genera mapping (Kim *et al.* 2019).

#### Functional annotation of lamprey genes and linking database resources

*Somatic annotation:* Of the 22,297 genes with PMZ IDs at UCSC, 4993 included putative gene names. To improve the functional annotation of the somatic genome, we employed the Trinotate pipeline. Using the fastA file containing all transcripts, the pipeline generated candidate gene names and/or gene ontologies for 11,284 genes. Of these, 3123 were also assigned names by Smith *et al.* (2018), and the Smith annotation file included gene names for an additional 1512 lamprey somatic genes; thus, of the 22,297 protein coding genes in the somatic assembly, we assigned names to 12,796 genes (57%). To allow comparison between the gene identifiers at the Ensembl, UCSC, and the Trinotate annotations generated here, we merged the three sources of information (Ensembl, UCSC, and Trinotate) to create a comprehensive reference database of gene identifiers and candidate gene names and gene ontologies of genes currently annotated in the lamprey somatic genome (Supplementary Table S4).

*Germline annotation:* Implementation of the Trinotate pipeline to the germline transcript file, generated 13,870 gene names and/or associated gene ontologies terms; of these, 12,990 were already annotated, and 880 generated new *ab initio* IDs. The majority of the overlapping IDs were congruent, and where discrepancies arose, it was often caused by synonymous gene names (Supplementary Table S5). Other recent transcriptomic studies performed in the sea lamprey have employed reference guided *de novo* assembly of transcripts (Hockman *et al.* 2019; Jones *et al.* 2019). For example, Hockman *et al.* (2019) first aligned reads to the sea lamprey reference germline genome available at SIMRbase, and then employed TRINITY to assemble *de novo* transcripts. Of the ∼120,000 transcripts they identified in embryonic samples, approximately half did not overlap with any annotated genes in the SIMRbase annotation file, indicating that there are still many new loci to be discovered in the lamprey genome.

The superiority of the germline reference genome, even for analyses based on somatic tissues, is such that it is recommended to use the germline assembly in studies moving forward. Nevertheless, the sea lamprey somatic reference genome and annotation have been hosted at Ensembl for nearly a decade, and served as the primary resource for lamprey phylogenetic and experimental studies. Thus, to help the lamprey genomics community make better use of the superior germline genome, we collected data on the gene ID’s, names, and ontologies for both the somatic and the germline reference genomes from UCSC, SIMRbase, and/or Ensembl and integrated them with gene ontology data obtained in this study and generated a database for cross-referencing (Supplementary Tables S4–S5). We next performed a blast-all against all search to compare all of the publicly available transcripts for the somatic (at UCSC) against those in the germline (at SIMRbase) transcriptomes. This generated 24,868 matched alignments with bit scores >50 (Supplementary Table S6). This data can be used to help link genes of interest annotated in the somatic P_marinusv. 7.0 genome available at UCSC with those for the germline assembly at SIMRbase, as well as to link these gene ID’s to those available at Ensembl (using Supplementary Tables S4–S5).

### Analysis of differentially expressed genes (DEG’s) in lamprey gonads

**Figure 3:**
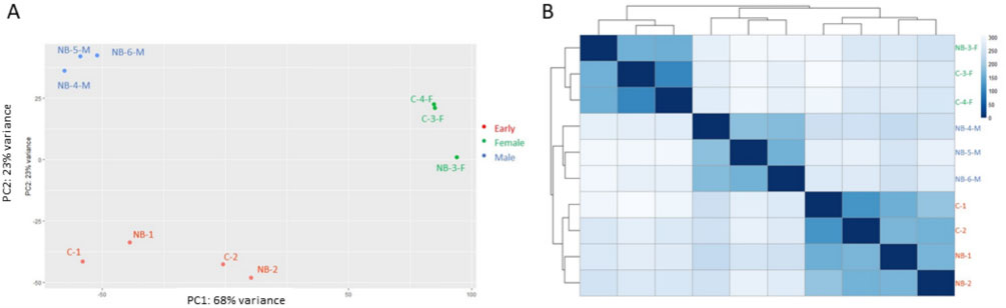
Relationship of global gene expression in the gonads of chestnut lamprey (C) and northern brook lamprey (NB) sampled in the early stages of differentiation (C-1, C-2, NB-1, NB-2) and in definitive females (C-3-F, C-4-F, NB-3-F) and males (NB-4-M, NB-5-M, NB-6-M) visualized with (A) a heat map of the sample-to-sample distance or (B) Principal Component Analysis as implemented in R platform 4.0.2 version of DESeq2.

Global gene expression profiles depicted in the unsupervised heat map and PCA plots were remarkably similar for gonads sampled at the same developmental stage, regardless of species (Figure 3). Insufficient replicates prevented between-species comparisons for differentiated females and differentiated males, so we cannot rule out species-specific differences in gene expression profiles when the two developmental trajectories diverge dramatically at metamorphosis. In the early stages of differentiation, we had two chestnut and two northern brook lampreys, but the gonads of C-1 and NB-1 included cysts of undifferentiated germ cells following the onset of mitosis while C-2 and NB-2 were in the early stages of meiosis, and global gene expression profiles corresponded with histological appearance of the gonad rather than species. Likewise, using a strict FDR of <0.01, only 81 genes were differentially expressed in parasitic (C-1 and C-2) *versus* nonparasitic (NB-1 and NB-2) lampreys (Supplementary Table S7A). None of these DEG’s appear related to gonadal development, nor do any correspond with genes that Spice *et al.* (2014) found were differentially expressed in these two species using a candidate gene approach and larger sample size. Spice *et al.* (2014) found that *igf1r* (insulin-like growth factor 1 receptor) expression was almost 100× higher in chestnut lamprey relative to northern brook lamprey during the first phase of oocyte growth, and *coIII* (cytochrome c oxidase subunit III) expression was 54–70× higher in the northern brook lamprey compared to the chestnut lamprey during all stages of development. Although more differences might have been expected given the general observation that nonparasitic species initiate ovarian differentiation earlier than parasitic lampreys, and usually produce fewer oocytes even during the larval stage (see Docker *et al.* 2019), this is not always the case. Spice and Docker (2014) found no significant difference in the timing of ovarian differentiation between northern brook and chestnut lampreys in Manitoba, and oocyte counts were similar in both species. However, more research with a greater number of biological replicates for each specific stage of early gonadal development is required to better understand differences in gene expression in parasitic *versus* nonparasitic lampreys.

**Figure 4:**
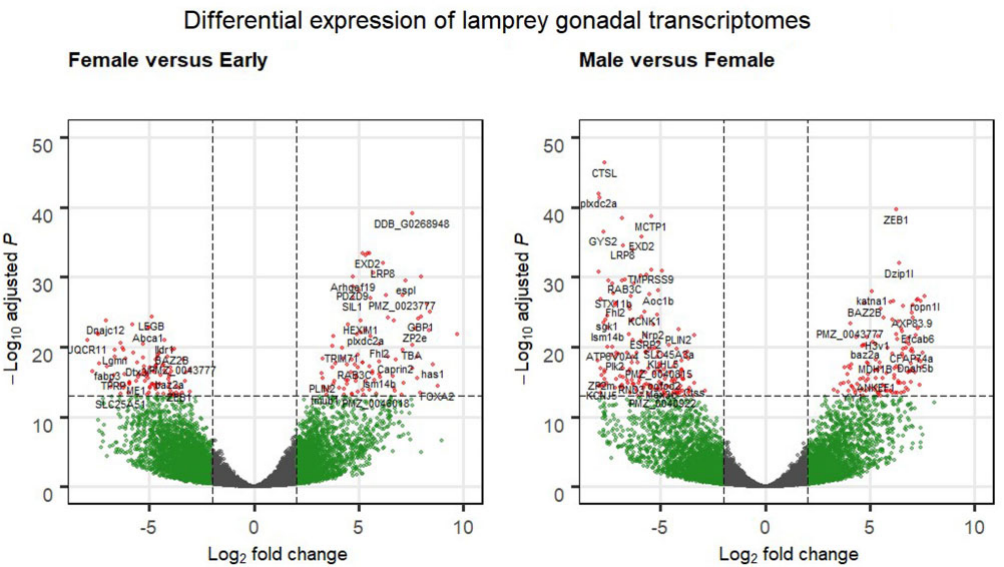
Volcano Plot of differentially expressed up- and down-regulated genes in the gonads of chestnut (C) and northern brook (NB) lamprey. (A) Definitive females *versus* larvae in the early stages of gonadal differentiation (C-1, C-2, NB-1, NB-2). (B) Males (NB-4-M, NB-5-M, NB-6-M) *versus* definitive females (C-3-F, C-4-F, NB-3-F), highlighting genes with a fold-change > log2 (four-fold differences) and *P* < 10^−14^. Details of which genes are up- and down-regulated in each analysis using a FDR of < 0.01 are given in Supplementary Table S7.

In contrast, using a strict FDR of <0.01, 2749 genes were more highly expressed in the gonads of larvae during early differentiation compared to 2308 genes in differentiated females (Figure 4A, Supplementary Table S7B), and 2645 *vs* 2842 genes were found to be significantly more highly expressed in the gonads of differentiated males *versus* differentiated females (Figure 4B, Supplementary Table S7C). Similarly high numbers of DEG’s have been identified following transcriptomic analyses of gonad development in other taxa (Piprek *et al.* 2019; Biscotti *et al.* 2020; Chiu *et al.* 2020) and in studies of sea lamprey development and environmental tolerance (Hockman *et al.* 2019; Jones *et al.* 2019).

#### Pathways enrichment and DEGs

**Figure 5:**
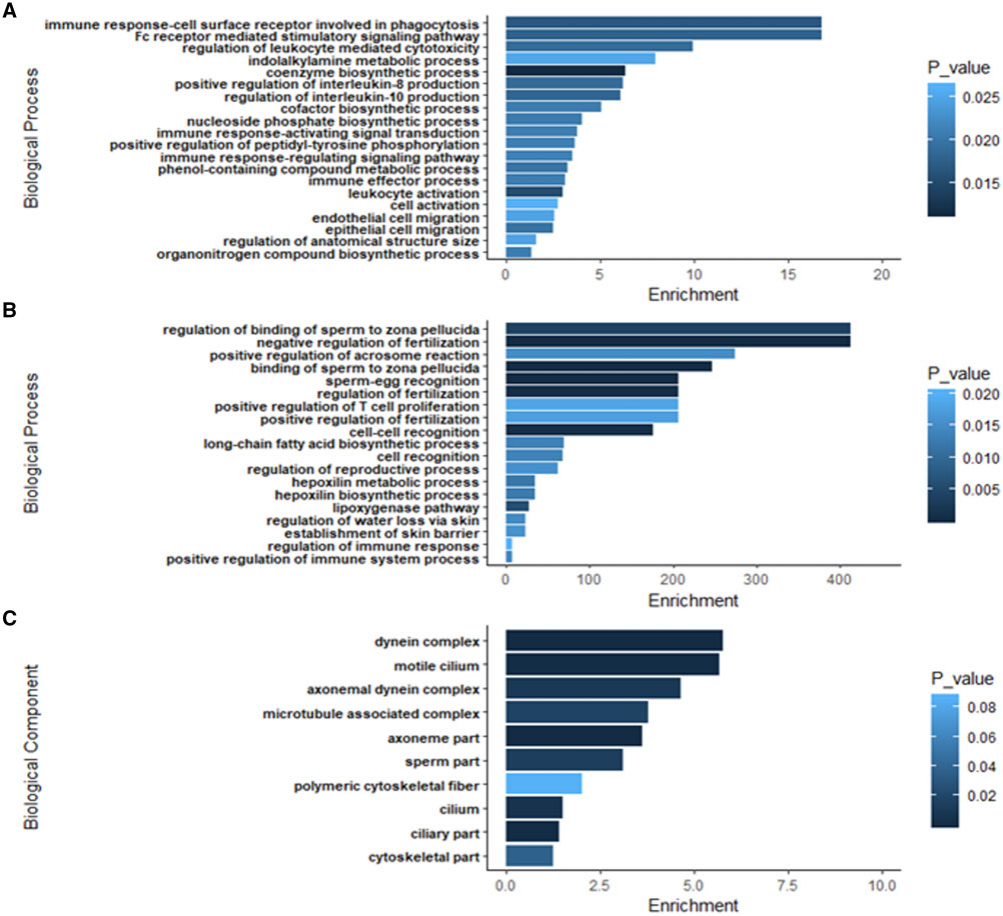
Histogram depicting enrichment for the top GO terms for (A) biological processes in chestnut and northern brook lamprey gonads in the early stages of differentiation, (B) biological processes in ovaries of definitive females, and (C) biological component for differentiated testes in males. Horizontal bars for each term are coloured by the FDR *P*-value for the association (see text).

**Figure 6:**
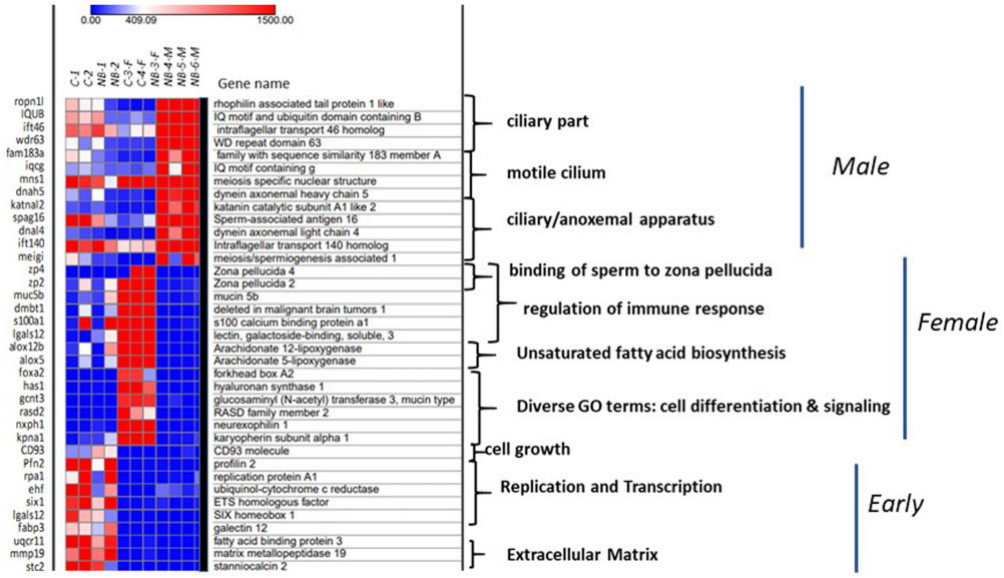
Heat map showing the normalised counts for 37 genes associated with GO term enriched pathways in gonads of chestnut and northern brook lampreys in the early stages of gonadal differentiation (C-1, C-2, NB-1, NB-2) and in definitive ovaries (C-3-F, C-4-F, NB-3-F) and testes (NB-4-M, NB-5-M, NB-6-M). Genes were identified with each of the GO terms (see Supplementary Table S8) and normalised counts extracted from the DESeq2 output and visualized across all samples using a logarithmic scale.

*Early differentiation:* In the four larval lamprey gonads undergoing mitotic proliferation (C-1, NB-1) or the early stages of meiosis I (C-2, NB-2), many of the upregulated pathways were associated with developmental and immune processes including: endothelial cell migration, indolakylamine metabolic process, nucleoside phosphate biosynthetic process, Fc receptor mediated stimulatory signaling pathways involving phagocytosis, as well as multiple immune pathways (positive regulation of IL-8 and IL-10), immune response-activating signal transduction among others (Figure 5A, Supplementary Table S7B). Closer examination of the log fold changes for some of the genes involved in these processes identified that a variety of genes associated with core processes of replication and transcription such as *pfn2, rpa1*, and *ehf*, were highly expressed during the early stages of gonadal differentiation (Figure 6). Genes associated with construction of the extracellular matrix, such as *mmp19* and *stc2*, were also highly expressed, while all of these genes were expressed at low or null levels in differentiated females and males (Figure 6).

*Differentiated females:* On the other hand, the ovaries of definitive females were highly enriched (Enrichment values >200) for processes related to regulation of fertilization including: binding to the zona pellucida, the acrosomal reaction, sperm–egg recognition, as well as cell–cell recognition. This is particularly interesting because, in these individuals, the oocytes were still undergoing slow cytoplasmic growth, and sexual maturation would not have occurred for at least another year (Figure 1). Additionally, differentiated females exhibited upregulation of pathways related to the regulation of immune responses, as well as biosynthesis of fats (long-chain fatty acid biosynthesis, lipoxygenase pathways, and hepoxilin biosynthetic process) (Figure 5B, Supplementary Table S7B). Collectively, this gives a strong biomolecular signature of vitellogenesis. Work in diverse, fish species has identified that vitellogenesis is typically initiated by binding of the steroid hormone 17β-estradiol (E_2_) to receptors in the liver and gonads. For example, in the liver, stimulation by E_2_ leads to upregulation of hepatically expressed genes such as: vitellogenin (*vtg*), the estrogen receptor 1 (*esr1*), insulin-like growth factor 1 (*igf1*), zona pellucida glycoproteins (zp’s), choriogenin H, and cytochrome p450s (Bon *et al.* 1997; Modig *et al.* 2006; Levi *et al.* 2009). Vitellogenesis also involves biosynthesis of fatty acids and steroids which would constitute components of the yolk, for example, and many of these are also derived from the liver in response to binding of E_2 _(Levi *et al.* 2009). In our DEG analyses, we find very high expression of zp in the gonads of our differentiated females (discussed below). Further, while we did not identify *esr1*, we found that *esrrb* was on an average 4.8-fold more expressed in female than male gonads (FDR = 1.2 × 10^–5^) (Supplementary Table S7C). We did not identify differential expression of igf1, which often emanates from the liver, but we did find that igf1r was 4.7-fold more highly expressed in differentiated females compared to larvae in the early stages of differentiation (FDR = 4.7 × 10^–6^), and that two *igf* binding proteins (*igfbp3* and *igfbp5*) were upregulated in ovaries compared to testes (Supplementary Table S7C).

Inspection of the genes underlying enriched pathways in differentiated females indicates that many of the GO terms are associated with zona pellucida glycoprotein (Supplementary Table S8). We extracted the normalized counts of gene expression of *zp2* and *zp4* from the DESeq2 output for all samples, and found that *zp4* is highly expressed in two of the three differentiated females (C-3-F and NB-3-F), but not in the males or larvae in the early stages of differentiation, while *zp2* was highly expressed in all three females, and was present in low levels in the two larvae in the early stages of meiosis I (C-2, NB-2) (Figure 6). These two individuals are likely presumptive females, although meiosis also produces primary oocytes in at least some future male lampreys (Docker *et al.* 2019). Similarly, the genes *mucin5B* and *dmbt1*, which are associated with activation of immune regulation, and arachidonate lipoxygenase 5 and 12 (*alox5* and *alox12b*), which are associated with unsaturated fatty acid metabolism (Supplementary Table S8), were present in the three differentiated ovaries; these four genes were not present in males, and they were expressed at lower levels in one or both larvae in the early stages of meiosis I. Similar patterns were observed for other genes associated with the female-enriched pathways for cell signaling (Figure 6).

*Differentiated males:* Lastly, for our three male gonadal samples, we found strong evidence of DEG’s associated with spermiation. The GO terms associated with biological processes and biological components enriched in the males were almost all associated with cilia formation, presumably associated with sperm flagella formation (Supplementary Table S8)(Ciereszko *et al.* 2002). For biological processes, three of the enriched terms were associated with cilia, two with microtubules/dynein-based processes (*e.g.*, involved in sperm motility), and the remaining ones for vesicle transport (Supplementary Table S8). For the GO Functional Component, we found enrichment for the same terms (ciliary, anoneme, and dynein), and examination of the genes underlying these pathways (Figure 6) shows strong enrichment for genes such as *iqub, wdr63, iqcg, mns1, dnah5, dna14*, and *meigi* that are predominantly testes-associated in other animals (*e.g.*, in humans; www.gtexportal.org). Some of these genes (particularly *iqub* and *mns1*) were also expressed in early differentiating larval gonads (including presumptive ovaries), although often at lower levels. In lampreys, both future sexes appear to pass through an initial female and an intersexual stage (Docker *et al.* 2019), and expression of these genes may reflect plasticity of initial gonadal development or be early markers of male development. Nevertheless, given that many of these genes have putative homologs in humans, it suggests that at least some of the genes involved in spermatogenesis are shared between lampreys and later diverging vertebrates. This will be examined in more detail in future work, by sampling more extensively across a greater number of gonadal stages, clarifying syntenic relationships among genes, and examining functional gene expression in further detail.

As a final note, it is important to caution that the putative functions and gene names associated with transcripts identified here are only starting points for further work in lampreys. Most of the gene names currently available for the sea lamprey germline genome are based on automated gene calling from SwissProt or UniProt (via MAKER or Trinotate, see above) and assign a single human or mouse ortholog for each lamprey gene. However, lampreys are estimated to have diverged from the vertebrate lineage after 1R (not 2R; Smith *et al.* 2018; Simakov *et al.* 2020), which would mean that there would not be a 1:1 orthology between genes in lampreys and 2R taxa, such as humans. Furthermore, in addition to being a putatively 1R taxon, the lamprey genome and other basal vertebrate lineages are thought to have undergone multiple independent chromosomal and/or region-specific duplications which would lead to lineage-specific amplification of some paralogous gene families (Smith *et al.* 2018). As a reflection of these difficulties, recent studies employing transcriptomic analyses in the sea lamprey have employed genome guided, *de novo* transcript assembly and discovered many genes that are not present in the current annotation (Hockman *et al.* 2019; Jones *et al.* 2019). Secondly, linking specific pathways or modes of function with genes by the general descriptors provided by GO is problematic due to the incomplete annotation of the sea lamprey genome and reliance on functional annotations from well-characterised taxa, although this approach is common when working in non-model vertebrates, including lampreys (Hockman *et al.* 2019). We chose human for the GO reference database, since the functional annotation of the human genome is among the most reliable; given the ancient divergence of humans (gnathostomes) from lampreys (agnathans) >500 mya, such analyses are expected to provide a conservative perspective on the likely function of lamprey genes (Smith *et al.* 2018).

In conclusion, in this paper, we have identified that the lamprey germline genome provides a much-improved reference for genome guided RNA-sequence analyses in lampreys, but that the current annotation of the genome requires more detailed work. Despite these difficulties, we identified many genes that are associated with gonadal development in lampreys, and, in particular, found suites of genes that appear to be expressed in a sex- or stage-specific manner. In future work, samples from a larger number of males and females sampled across a broader range of developmental stages will be important to test for sex-specific gene expression in the lamprey gonad. In particular, the current study did not include any individuals approaching sexual maturation; females were all pre-vitellogenic and males were still undergoing spermatogonial (mitotic) proliferation (Figure 1). Furthermore, although we detected few (and arguably no biologically meaningful) differences between the parasitic chestnut lamprey and the nonparasitic northern brook lamprey given the lack of true biological replicates, more work and larger sample sizes is needed to test for species-specific differences in gene expression profiles, especially when the two developmental trajectories diverge more dramatically following metamorphosis. Finally, future work will be needed to perform the requisite detailed phylogenetic, syntenic, and expression analyses to establish the orthology and the comparative role of these genes with those in higher vertebrates.

## Data availability

The RNA-sequencing reads are available in the NCBI Sequence Read Archive under BioProject PRJNA691994. Supplementary Tables S1–S6 consist of tables describing mapping statistics to the somatic genome (Table S1), gene counts of the reads to the somatic (Table S2) and the germline (Table S3) reference genomes, gene annotation tables for the somatic (Table S4) and the germline (Table S5) genomes as well as the blast-all-against-all to compare transcripts in the two genomes (Table S6). Table S7 contain lists of differentially expressed genes (DEG’s) for comparisons between species and gonadal stage. Table S8 contains the GO-terms associated with DEG’s for larval, male, and female biological processes. Supplementary Material available at figshare: https://doi.org/10.25387/g3.13514007.
